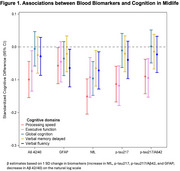# Alzheimer disease blood biomarkers and cognition in midlife

**DOI:** 10.1002/alz70856_101201

**Published:** 2025-12-25

**Authors:** Xiaqing Jiang, Tina D Hoang, Leslie M. Shaw, David R Jacobs, Ilya M. Nasrallah, R Nick Bryan, Kristine Yaffe

**Affiliations:** ^1^ University of California, San Francisco, San Francisco, CA, USA; ^2^ NCIRE‐The Veterans Health Research Institute, San Francisco, CA, USA; ^3^ Perelman School of Medicine, University of Pennsylvania, Philadelphia, PA, USA; ^4^ University of Minnesota, Minneapolis, MN, USA; ^5^ University of Pennsylvania, Philadelphia, PA, USA; ^6^ University of Pennsylvania Health System, Philadeplphia, PA, USA

## Abstract

**Background:**

Despite a surge in studies on Alzheimer disease (AD) blood biomarkers, most evidence comes from clinical samples of older adults. The relationship between blood biomarkers and cognition in midlife remains poorly understood.

**Method:**

We cross‐sectionally studied 1,356 dementia‐free Black and White participants (58% women and 45% Black), enrolled in the Coronary Artery Risk Development in Young Adults Study (CARDIA) with a mean age of 61±3.6 (range 53‐69) years. Five cognitive tests assessing different domains were administered. Plasma β‐amyloid (Aβ) 42/40 and phosphorylated tau (*p*‐tau)217 were assayed using the Fujirebio Lumipulse G1200 analyzer, while glial fibrillary acidic protein (GFAP) and neurofilament light chain (NfL) were analyzed using the Quanterix Simoa HD‐X assay. Associations of demographic and clinical characteristics with plasma biomarker levels (log‐transformed) were evaluated using linear regression. We also used linear regression to assess the standardized association between plasma biomarker level (per 1 SD) and midlife cognition, testing for effect modification by race.

**Result:**

Older age, White race, *APOE ε4* carrier, and diabetes were associated with biomarker levels indicative of higher AD risk (lower Aβ42/40; higher *p*‐tau217, *p*‐tau217/Aβ42, NfL, and GFAP). After adjustment for age, sex, race, education, body mass index, and estimated glomerular filtration rate, lower Aβ42/40, higher *p*‐tau217, and higher *p*‐tau217/Aβ42 were each associated with worse performance on processing speed and executive function (Figure 1). Higher NfL was associated with worse performance on all five domains, while higher GFAP was associated with worse performance on processing speed and verbal fluency. These associations remained significant after additional adjustment for *APOE* and cardiovascular risk factors, with no clear pattern for race interaction in biomarker‐cognition associations.

**Conclusion:**

AD pathology and neurodegeneration indicated by blood biomarkers may already start contributing to cognitive function in midlife. Young and middle adulthood is a critical time for AD prevention, which may delay the onset of cognitive decline and dementia among middle‐aged and older adults.